# Supplementary material for: Active site specificity profiling datasets of matrix metalloproteinases (MMPs) 1, 2, 3, 7, 8, 9, 12, 13 and 14
Source: Data Brief. 2016 Feb 22;7:299–310. doi: 10.1016/j.dib.2016.02.036 (PMC4777984; doi:10.1016/j.dib.2016.02.036)
Supplement: Supplementary file 10 — Supplementary material [file mmc10.zip › WebPICS_hMMP8_T_1%/P1prime.html]

 

PICS results


|  |  |
| --- | --- |
| **P1prime\_C**  17 in 335 sites   5.1 %    effects > 10 perc. pnts.  (vice-versa in brackets)  P2prime\_H: 14.0 (19.9) |  |
| **P1prime\_I**  52 in 335 sites   15.5 %    effects > 10 perc. pnts.  (vice-versa in brackets)  P2prime\_L: 16.7 (14.5) |  |
| **P1prime\_V**  32 in 335 sites   9.6 %    effects > 10 perc. pnts.  (vice-versa in brackets)  P2\_Q: 10.7 (12.6) |  |
| **P1prime\_W**  7 in 335 sites   2.1 %    effects > 10 perc. pnts.  (vice-versa in brackets)  P1\_G: 48.1 (11.2) |  |
| **P1prime\_Y**  22 in 335 sites   6.6 %    effects > 10 perc. pnts.  (vice-versa in brackets)  P2\_E: 14.0 (10.6) |  |
